# Erector Spinae Plane Block for Elective Laparoscopic Cholecystectomy in the Ambulatory Surgical Setting

**DOI:** 10.1155/2018/5492527

**Published:** 2018-04-01

**Authors:** Kjartan Eskjaer Hannig, Christian Jessen, Uday Kant Soni, Jens Børglum, Thomas Fichtner Bendtsen

**Affiliations:** ^1^Department of Anesthesiology, Horsens Hospital, Sundvej 30, 8700 Horsens, Denmark; ^2^Department of Anesthesiology, Zealand University Hospital, University of Copenhagen, Copenhagen, Denmark; ^3^Department of Anesthesiology, Aarhus University Hospital, Aarhus, Denmark

## Abstract

Postoperative pain after laparoscopic cholecystectomy can be severe. Despite multimodal analgesia regimes, administration of high doses of opioids is often necessary. This can further lead to several adverse effects such as drowsiness and respiratory impairment as well as postoperative nausea and vomiting. This will hinder early mobilization and discharge of the patient from the day surgery setting and is suboptimal in an Early Recovery after Surgery setting. The ultrasound-guided Erector Spinae Plane (ESP) block is a novel truncal interfascial block technique providing analgesia of the thoracic or abdominal segmental innervation depending on the level of administration. Local anesthetic penetrates anteriorly presumably through the costotransverse foramina to the paravertebral space. We demonstrate the analgesic efficacy of the ESP block in a case series of three patients scheduled for ambulatory laparoscopic cholecystectomy.

## 1. Introduction

Laparoscopic cholecystectomy is associated with moderate to severe pain despite all currently available multimodal analgesic regimes [1–3]. The ultrasound-guided Erector Spinae Plane (ESP) block is a novel truncal interfascial regional technique [4]. Two variations of the ESP block for thoracic and abdominal procedures have recently been described in literature. The injection site is either at the level of T5 transverse process, resulting in spread between the C7 and T8 segmental levels [4–6], or at the levels of the T7-T9 transverse processes, resulting in spread between the T6 and T12 segmental levels [7–9]. Local anesthetic penetrates anteriorly presumably through the costotransverse foramina to the paravertebral space and it can thus be described as an indirect paravertebral block [8]. Several approaches targeting the same interfascial plane have been described in the current literature with variable injection sites [10, 11]. However, the ESP block is presumably the most promising due to its anatomically close proximity to the costotransverse foramina.

## 2. Case Presentation

Written informed consent for publication was obtained from all three patients. There was no need for approval by the Central Denmark Region Committees on Biomedical Research Ethics.

### 2.1. Patient 1

**Figure 1:**
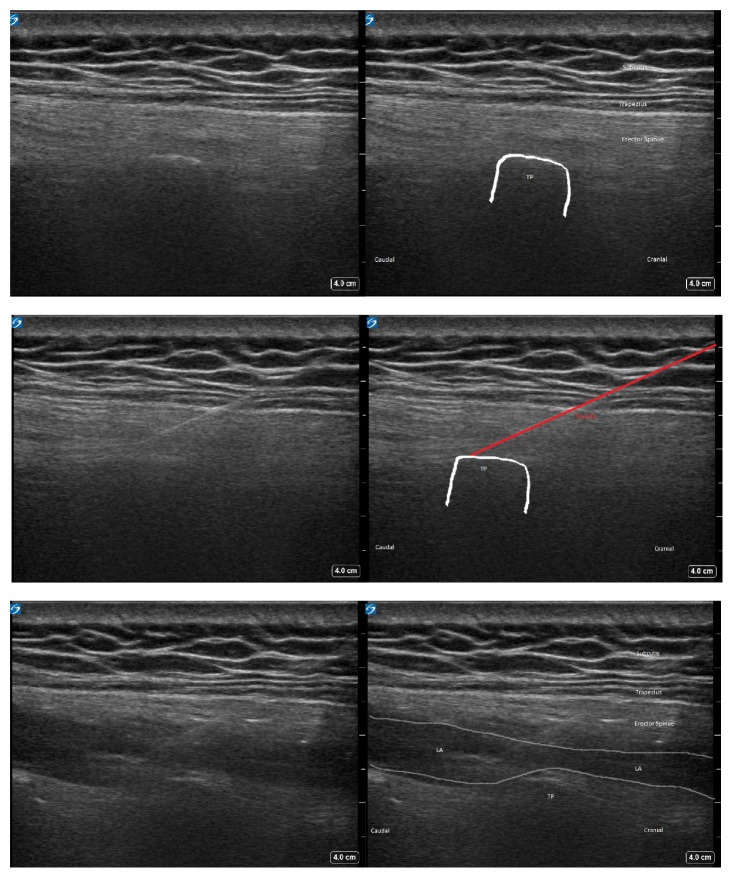
Ultrasound image showing subcutis, trapezius muscle, erector spinae muscle, transverse process of T7 (TP), needle path (in red), and spread of local anesthetic (LA).

A 42-year-old man with a body mass index (BMI) of 35.8 kg/m^2^ presented for elective ambulatory laparoscopic cholecystectomy. He was diagnosed with obstructive sleep apnea treated with nightly continuous positive airway pressure (CPAP) therapy. He was otherwise healthy and did not take any medication. A bilateral ESP block was performed just prior to surgery. The patient was sitting up and the level of the T7 transverse process was located just 3 cm laterally from the midline using a 15–6 MHz linear ultrasound probe (SonoSite, X-Porte, Bothell, Washington) oriented sagittally (Figure 1). The cross-sectional view of the transverse process was centered on the ultrasound screen and the overlying trapezius, and erector spinae muscles were identified. Under aseptic conditions, a 22-gauge 80 mm block needle (Pajunk, Geisingen, Germany) was inserted in-plane at a shallow angle of approximately 30–40° in a cranial-to-caudal direction aiming at the needle tip at the posterior aspect of the transverse process. Bony contact was established, and hydrodissection with 2 mL of isotone saline confirmed correct needle tip position on the transverse process and deep to the epimysium of the erector spinae muscle. The needle tip was then redirected and advanced slightly caudad to the transverse process. After hydrodissection with yet another 2 mL of isotonic saline, injection of 20 mL ropivacaine 0.5% was performed ensuring both cranial and caudal spread of local anesthetic in the fascial plane deep to the erector spinae muscle, lifting the muscle off the transverse processes. The procedure was repeated in a similar fashion on the contralateral side. Preoperative multimodal analgesia was achieved with paracetamol 1 g and ibuprofen 400 mg orally. General anesthesia was induced with propofol 180 mg and remifentanil 350 *μ*g and maintained with propofol 65 *μ*g/kg/min and remifentanil 0.33 *μ*g/kg/min. Intubation was uneventful. The patient received intraoperative dexamethasone 8 mg IV, droperidol 0.625 mg IV, and ondansetron 4 mg IV. Fentanyl 50 *μ*g IV was also administered intraoperatively 30 min prior to emergence from anesthesia. Pneumoperitoneum was achieved with insufflation of carbon dioxide (CO_2_) with a pressure of 12 mmHg. At the end of the uncomplicated surgical procedure the laparoscopic port sites were infiltrated with 20 mL of ropivacaine 0.2%, and CO_2_ was evacuated. Extubation was uneventful but in the postanesthesia care unit (PACU), the patient was drowsy and complained about dyspnea, which was suspected to be caused by a combination of mild intermittent airway obstruction, anxiety, and pain. It was thus treated with a nasal airway, fentanyl 50 *μ*g IV, and morphine 10 mg orally. Once fully awake, the patient denied that the incident was due to pain but reported that he had been anxious because of dyspnea. Numerical rating scale (NRS) was 2/10 and remained below 3/10 for the rest of his stay in the PACU and he did not receive any further medication. He was discharged to home 2 hours and 56 min after arriving in the PACU.

### 2.2. Patient 2

A 49-year-old woman (BMI 28.4 kg/m^2^) was scheduled for ambulatory laparoscopic cholecystectomy. She did not present any comorbidities besides being a smoker (40 pack years). The ESP block was performed 45 min preoperatively at the level of the T7 transverse process as described above under mild sedation with fentanyl 50 *μ*g IV. She received standard premedication with paracetamol and ibuprofen. Induction was performed with propofol 190 mg and remifentanil 250 *μ*g and maintained with propofol 70 *μ*g/kg/min and remifentanil 0.3 *μ*g/kg/min. Intubation and extubation were uneventful. Intraoperative medication was similar to patient 1. The first NRS in the PACU was 2/10 and subsequent scores were 0–2/10. Morphine 10 mg was administered orally due to earlier standard protocols, although the patient did not have any pain complaints. The patient was discharged to home from the PACU 3 hours and 32 min after arrival.

### 2.3. Patient 3

A 65-year-old woman (BMI 33.3 kg/m^2^) also presented for ambulatory laparoscopic cholecystectomy. Her only comorbidities were depression and anxiety, which were well controlled with oral nortriptyline and pregabalin. Premedication with paracetamol and ibuprofen was similar to the other two cases described above. The ESP block was carried out 45 min preoperatively at the level of the T7 transverse process similar to patients 1 and 2. Induction was achieved with propofol 140 mg and remifentanil 250 *μ*g and maintained with propofol 71 *μ*g/kg/min and remifentanil 0.13 *μ*g/kg/min. She also received intraoperative dexamethasone, droperidol, ondansetron, and port site infiltration. Fentanyl 100 *μ*g was administered intraoperatively. After uncomplicated surgery and extubation the patient was transferred to the PACU. In the PACU the patient only complained of minor pain (NRS < 3/10). She received fentanyl 50 *μ*g, and at discharge to home 2 hours and 1 min later her NRS score was 0/10.

Data on all three cases are summarized in Table 1.

## 3. Discussion

In developed countries, approximately 15% of asymptomatic adults from the general population have gallstones, and nearly 10% of these individuals develop symptoms or complications requiring treatment within five years [12]. Cholecystectomy is the golden standard in treating symptomatic gallbladder disease, such as acute cholecystitis [12]. In Denmark, about 9000 cholecystectomies are performed annually, and 93% of these are performed as laparoscopic procedures [13]. Currently, about 40% of the laparoscopic cholecystectomies are performed as ambulatory surgery [13], and approximately 15% of the ambulatory patients cannot be discharged on the day of the surgery most often due to pain or postoperative nausea and vomiting (PONV) [12].

Pain can be severe after laparoscopic cholecystectomy [2, 3]. This pain has three components: incisional pain (somatic pain from the trocar site), visceral pain, and shoulder pain (presumably referred visceral pain) [2, 3]. Pain in general is most intense on the day of the surgery (peaking within the first 4–8 hours after surgery) and subsequently diminishes to low levels within 2–4 days [2, 3, 14]. The intensity of visceral pain dominates over incisional pain (especially periumbilical), which dominates over shoulder pain [2]. Three possible mechanisms for visceral pain have been proposed: (1) irritative effect on the diaphragmatic peritoneum of the insufflated CO_2_ gas because of conversion to carbonic acid, (2) diaphragmatic muscle fiber stretching with tearing of muscular blood vessels and traction on nerve fibers, and (3) retained residual pockets of gas in the abdominal cavity [2, 3, 15]. About 30–50% of patients suffer from shoulder pain during the first postoperative day, most often on the right side [15]. It is usually of short duration and low intensity [2, 15]. The mechanism of shoulder pain is multifactorial and poorly understood, but it is thought to be subdiaphragmatic irritation transmitted via the phrenic nerve causing referred pain in the C4 dermatome [2, 15].

For comparison, regarding how the normal patient without a block does at our institution, we have carried out a survey from the first 50 laparoscopic cholecystectomies at our department in 2016 (yet unpublished data) with multimodal analgesia (paracetamol, ibuprofen, dexamethasone, port site infiltration, and opioids as rescue medication). Immediately after awakening, about half of patients (56%) had minor pain (NRS 0–3) and about half of patients (44%) had moderate to severe pain (NRS 4–10). About one-fourth of the patients (24%) just had minor pain (NRS 0–3) during the entire PACU stay, whereas about three-fourths of the patients (76%) at some point had moderate to severe pain (NRS 4–10). Interestingly, only half of the patients who initially just had minor pain (NRS 0–3) continued having minor pain, whereas the other half of the patients with initial minor pain experienced stronger pain later during the PACU stay. Another point of interest is that 1 out of 7 patients (14%) at some point in the PACU had excruciating pain (NRS 8–10), which corresponds to earlier reported numbers [16]. The patients received on average 47 mg morphine (oral equivalents) intraoperatively (standard deviation 14.8) and 45 mg postoperatively (SD 31.8) during the PACU stay. Prevalence of PONV in the PACU was 40%, requiring additional pharmacological treatment besides the administered standard triple prophylaxis. The average discharge time was 3.5 hours (SD 1.4), and 20% were admitted overnight most often due to pain. The clinical course of patients discharged after ambulatory laparoscopic cholecystectomy (without block) in our department has been previously described [14]. Median pain intensity was NRS 7 on the day of surgery and NRS 6, 5, 4, 1 on the 1st, 2nd, 3rd, 7th postoperative day, respectively [14]. After discharge 27.9% of the patients had unscheduled pain-related contacts with healthcare services, most often in the 1st postoperative week [14].

Opioid requirements at home after ambulatory laparoscopic cholecystectomy are variable [17]. According to Hill et al. about 35% of discharged patients did not take opioids, about 25% took 1–10 pills of oxycodone 5 mg (equivalent to 7.5 mg oral morphine), about 25% took 11–15 pills, and about 15% took more than 15 pills [17].

PONV has previously been described to exist in about 10% of patients after laparoscopic cholecystectomy with multimodal anesthesia [2, 16]. Pain is strongly associated with PONV [16]. Pain may directly worsen PONV, and PONV in itself may aggravate the pain [16].

The concept of “fast-track surgery” or Enhanced Recovery after Surgery (ERAS) programs applied on laparoscopic cholecystectomy requires multimodal analgesia including paracetamol, NSAIDs and dexamethasone in addition to opioids as rescue medication [1–3, 18]. Port site local infiltration has been shown to provide some pain relief, as opposed to intraperitoneal local anesthetic [3, 19]. Low pressure pneumoperitoneum (<10 mmHg) has been shown to decrease pain scores and analgesic consumption as opposed to standard pressure (12–16 mmHg) [3, 15, 20]. Active gas aspiration and pulmonary recruitment maneuvers after the completion of the surgical procedure empty the abdominal cavity from residual CO_2_ gas and reduce postoperative overall pain [15, 21]. This is especially true for shoulder pain and possibly also for (upper) abdominal pain [21]. Classical lateral Transversus Abdominis Plane (TAP) blocks have shown marginal benefit in reducing opioid consumption and pain scores in the first 6–8 hours similar to that of local port site infiltration [19, 22]. TAP blocks only provide somatic analgesia [19].

Since its first description in 2016 numerous case reports about the ESP block have been described for thoracic and abdominal procedures including thoracic neuropathic pain [4, 6], ventral hernia repair [7], bariatric surgery [8], thoracotomy with lobectomy [5], rib tumor surgery [23], and major lower abdominal surgery [9].

Unlike earlier beliefs, the fascial planes in the paravertebral region may not be bound true compartments [24]. Local anesthetic seems to spread along the dorsal rami of the thoracic spinal nerves, which run through the costotransverse foramina of Cruveilhier. Ligaments like the superior costotransverse ligament seem to have fenestrations [24], and these porous anatomical structures can be speculated to allow anterior spread of the local anesthetic. The needle tip positioning for the ESP block is technically simple, safe, and away from the paravertebral space and the pleura. The ESP block injectate reaches the dorsal and ventral rami of the thoracic spinal nerves as well as the sympathetic gray communicating rami [4, 7, 23]. The ESP block generates extensive blockade of the posterior, lateral, and anterior thoracic and abdominal wall and thus alleviates incisional pain. In addition, the ESP blocks visceral autonomic pain [6, 8].

There are several unanswered questions to address. Firstly, the ESP block has so far only been described in case reports, and the promising results must be validated in future randomized clinical trials. Secondly, the optimal time for block placement should be considered. In general, this is the best achieved preoperatively in the awake patient. About three-fourths of the patients experience moderate to severe pain some time during the postoperative period. A minority of the patients experience excruciating pain. Thirdly, optimal volume and concentration of local anesthetic are unknown. Previous authors have mainly used ropivacaine 0.5% 20 mL providing analgesia for about 20 hours reducing opioid consumption to about one-third [7]. A similar reduction from the expected opioid usage was seen in our three cases (see Table 1). The opioid sparing potential may be especially advantageous in the ambulatory setting, where pain and/or PONV may delay or even prevent same-day discharge. Fourthly, additives like glucocorticosteroids (off-label use) can be considered [7], which presumably would extend block duration beyond 24 hours.

## 4. Conclusion

The three reported cases illustrate the efficacy of the ESP block for somatic and visceral pain relief after upper abdominal laparoscopic cholecystectomy. The results must be validated in future randomized controlled trials.

## Figures and Tables

**Table 1:** Data from all three patients on opioid consumption, pain scores, and other variables.

	Patient 1	Patient 2	Patient 3
Discomfort with block placement (0–10)	1	0	4
In-hospital opioids^*∗*^			
*Intraoperative (during surgery)*	15 mg	15 mg	15 mg
* Postoperative (in PACU)*	25 mg	10 mg	15 mg
NRS in PACU (0–10)			
* First NRS*	2	2	3
* Highest NRS*	3	2	3
PONV in PACU	No	No	No
Time to discharge from PACU	2 h 56 min	3 h 32 min	2 h 1 min
Opioid requirements after discharge^*∗*^			
* 1st postoperative day*	10 mg	20 mg	20 mg
* 2nd postoperative day*	0 mg	10 mg	0 mg
* In total 1st week*	10 mg	40 mg	20 mg
Mean NRS after discharge (0–10)			
* 1st postoperative day*	4	3	4
* 2nd postoperative day*	3	2	2
* 7th postoperative day*	0	0	0
PDNV			
* 1st postoperative day*	No	No	No
* 2nd postoperative day*	No	No	No
Unscheduled healthcare contacts	No	No	No
(within 1st week after surgery)			
Resumption of ADL (days)	5	7	10

NRS: numerical rating scale. PACU: postanesthesia care unit. PDNV: postdischarge nausea and vomiting. ADL: activities of daily living.  ^*∗*^Oral morphine equivalents. PONV: postoperative nausea and vomiting.
